# Acanthoic Acid Can Partially Prevent Alcohol Exposure-Induced Liver Lipid Deposition and Inflammation

**DOI:** 10.3389/fphar.2017.00134

**Published:** 2017-03-16

**Authors:** You-Li Yao, Xin Han, Zhi-Man Li, Li-Hua Lian, Ji-Xing Nan, Yan-Ling Wu

**Affiliations:** ^1^Key Laboratory for Natural Resource of Changbai Mountain & Functional Molecules, Ministry of Education, College of Pharmacy, Yanbian University Yanji, China; ^2^Clinical Research Center, Yanbian University Hospital Yanji, China

**Keywords:** acanthoic acid, alcohol, liver lipid deposition, inflammation, Sirt1, LXRs

## Abstract

**Aims:** The present study aims to detect the effect of acanthoic acid (AA) on alcohol exposure-induced liver lipid deposition and inflammation, and to explore the mechanisms.

**Methods:** C57BL/6 mice were pretreated with single dose of AA (20 and 40 mg/kg) by oral gavage or equal volume of saline, and then exposed to three doses of ethanol (5 g/kg body weight, 25%, w/v) by gavage within 24 h. The mice were sacrificed at 6 h after the last ethanol dosing. Serum and hepatic indexes were detected by western blot, RT-PCR, and histopathological assay. AML-12 cells were pretreated with AA (5, 10, 20 μM), or AICAR (500 μM), GW3965 (1 μM), SRT1720 (6 μM), Nicotinamide (20 mM) for 2 h, respectively, and then following treated with EtOH (200 mM) and lipopolysaccharide (LPS) (10 ng/ml) for additional 48 h. Cell protein and mRNA were collected for western blot and RT-PCR. Cytokines interleukin-1β (IL-1β) and tumor necrosis factor-α (TNF-α) release were detected by ELISA assay.

**Results:** It was found that AA significantly decreased acute ethanol-induced increasing of the serum ALT/AST, LDH, ALP levels, and hepatic and serum triglyceride levels, and reduced fat droplets accumulation in mice liver. AA significantly suppressed the levels of sterol regulatory element binding protein 1 (SREBP-1), cytochrome P4502E1 (CYP2E1), IL-1β, and caspase-1 induced by ethanol. Furthermore, a significant decline of sirtuin 1 (Sirt1) and liver X receptors (LXRs) levels was observed in EtOH group, compared with normal group mice. And AA pretreatment increased the Sirt1 and LXRs levels, and also ameliorated phosphorylation of liver kinase B-1 (LKB-1), adenosine monophosphate-activated protein kinase (AMPK), acetyl CoA carboxylase (ACC) proteins, compared with EtOH group. However, the levels of peroxisome proliferator activated receptor -α or -γ (PPAR-α or PPAR-γ) induced by acute ethanol were reversed by AA. In EtOH/LPS cultivated AML-12 cells, AA decreased IL-1β and TNF-α levels, lipid droplets, and SREBP-1 and CYP2E1 expressions, compared with EtOH/LPS treatment. AA also significantly increased protein expressions of Sirt1, p-LKB1, p-ACC, PPARα, and decreased protein expression of PPARγ, compared with EtOH/LPS treatment.

**Conclusion:** Acanthoic acid can partially prevent alcohol exposure-induced liver lipid deposition and inflammation via regulation of LKB1/Sirt1/AMPK/ACC and LXRs pathways.

## Introduction

Alcohol consumption is the single most important factor for alcohol liver disease, and the latter is a major health problem all over the world. Alcoholic liver diseases (ALD) include simple fatty liver, steatosis or fibrosis, and more severe liver injury such as alcoholic hepatitis, cirrhosis, and hepatocellular carcinoma (Mathurin and Bataller, 2015; Addolorato et al., 2016). It is investigated that ALD is liable for 5.9% global mortality, 5.5% global disease burden and 2.5 million deaths worldwide (European Association for the Study of Liver, 2012; Dugum and McCullough, 2015). Fatty liver is the earliest stage and characterized by fat accumulation in hepatocytes. Some steatosis even progress to steatohepatitis and accompany the persistence of fatty liver and inflammation. In the later stage, collagen deposition and regenerative nodules would result in fibrosis and cirrhosis. Alcohol withdrawal is necessary to prevent the development of ALD. And also seeking the mechanisms of alcoholic liver damage may supply pharmacologic therapeutic strategies to reduce disease progression.

The liver is the major organ of alcohol metabolism and a main targeting tissue damaged by alcohol. Alcohol can induce the increasing of TG and free fatty acid synthesis, slow down the elimination of free fatty acid in hepatocytes, and break the equilibrium of synthesis and secretion of TG, eventually lead to excessive deposition of lipid in liver and form fatty liver. Another, inflammatory reaction is also the most important reason of ALD. Intestines intake alcohol and increase intestinal endotoxemia to enter into blood by enterohepatic circulation, and then further activate Kupffer cells to release inflammatory factors. Current researches have indicated that alcohol and its metabolites play important roles in the formation of alcoholic fatty liver through hepatotoxicity, oxidative stress, lipid peroxidation, and ethanol metabolic enzyme system. Although, the great development of ALD has been performed in recent years, the precise mechanisms are not well understood. In the current study, mice were exposed to three doses of ethanol (5 g/kg) by gavage with 12 h intervals. The acute ethanol mice model has been wildly used in many references (Carson and Pruett, 1996; Izu et al., 2006; Kumar et al., 2011; Chen et al., 2014). In order to elucidate the mechanism in the pathogenesis of acute ethanol-induced liver damage, this animal model was utilized for the evaluation *in vivo*. And the acute ethanol exposure induced inflammation and significantly increased LPS levels. Thus, ethanol was exposed to LPS-primed AML-12 cells to mimic hepatocytes development during ALD progression *in vitro.*

Sirtuin (silent mating type information regulation 2 homolog) 1 (Sirt1), a kind of nicotinamide (NAD^+^)-dependent protein deacetylase, may control fatty acid homeostasis in liver. Study has been done regarding in the partial absence of Sirt1 activity, even normal diet also can increase liver steatosis in Sirt1+/- mice, and possible mechanism is related with the increase of lipid synthesis in the liver of Sirt1+/- mice (Xu et al., 2010). It was reported the compounds from Acanthopanax upregulate Sirt1 gene expression (Park et al., 2015). Therefore, we consider whether Acanthoic acid (AA) has the same up-regulation effect on Sirt1.

**FIGURE 1:**
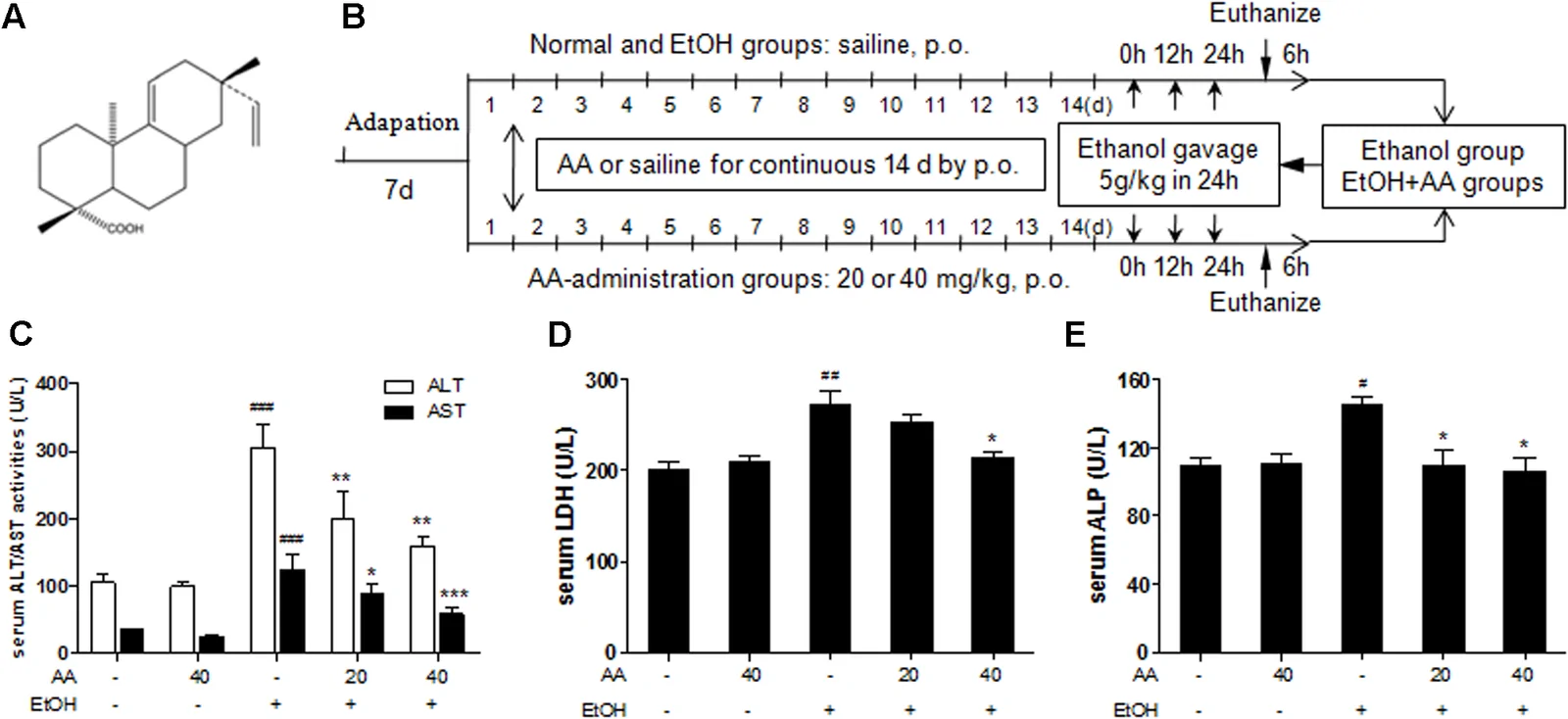
**Effects of acanthoic acid (AA) on the serum ALT/AST activities, the serum LDH, and ALP activities.** **(A)** Chemical structure of AA; **(B)** Methods of animals and ethanol treatment; **(C)** Serum ALT/AST activity; **(D)** serum LDH activity; **(E)** serum ALP activity. Data were presented as mean ± SD (*n* = 6). ^#^*p* < 0.05, ^##^*p* < 0.01,^###^*p* < 0.001 vs. normal group; ^∗^*p* < 0.05, ^∗∗^*p* < 0.01, ^∗∗∗^*p* < 0.001 vs. EtOH group.

Acanthoic acid (AA, (-)-primara-9(11), 15-dien-19-oic acid, **Figure 1A**) is a pimarane-type diterpene extracted from *Acanthopanax koreanum* Nakai (Araliaceae). This plant has been classified as a unique species of the *Acanthopanax* genus, and traditionally used in the treatment of rheumatism, diabetes, and hepatitis (Jung et al., 2013). AA exhibits various biological properties, including anti-inflammatory and antioxidant (Través et al., 2014; Wei et al., 2015). Our previous studies have indicated that AA shows a strong protective effect against drug-induced hepatotoxicity, and works as a LXRs agonist regulating hepatic fibrosis in mice (Wu et al., 2010; Bai et al., 2014). However, Sirt1 is a positive regulator of LXR proteins. Subsequently, the present study aims to identify the regulation effect of AA on alcohol exposure-induced liver lipid deposition and inflammation, and elucidate the expression of Sirt1 under AA treatment and the Sirt1 relative mechanism of AA treatment on alcohol exposure-induced liver lipid deposition and inflammation.

## Materials and Methods

### Plant Material

*Acanthopanax koreanum* Nakai (Araliaceae) was afforded by Yanbian University. A voucher specimen (YBUCP201001) was deposited in the Herbarium of College of Pharmacy, Yanbian University, China. AA was isolated from the roots of *Acanthopanax koreanum* Nakai (Araliaceae) as described previously (Kim et al., 1988; Bai et al., 2014), and obtained by a preparative HPLC with a yield of 0.82% and purity >95%.

### Animal Procedures

Male C57BL/6 mice (6-week-old; body weight 18–22 g) were obtained from Changchun Yisi Laboratory Animal Technology Co., Ltd (Jilin, People’s Republic of China) [SPF, SCXK (J) 2003-0008]. And all mice were maintained in controlled environment with temperature of 22 ± 2°C, 50–60% of relative humidity and a 12-h light, 12-h dark cycle per day during the experiment period. The experiment procedures were approved by the Institutional Animal Care and Use Committee of Yanbian University (Resolution number, 201501021).

Mice were randomly separated into the following five groups: normal group, AA-40 group, EtOH group, EtOH+AA-20 group, EtOH+AA-40 group, each group six mice. AA administration groups, including AA-40, EtOH+AA-20, EtOH+AA-40 groups, were pretreated with single dose of AA (20 or 40 mg/kg body weight, respectively) by oral gavage for 14 consecutive days. At the same time, normal group and EtOH group were administrated by gavage with equal volume of saline for 14 consecutive days. Following, except normal group and AA-40 group, other groups were exposed to three doses of ethanol (5 g/kg body weight) by gavage within 24 h (Portari et al., 2016; Yang et al., 2016). All animals were sacrificed at 6 h after the last ethanol dosing and blood was taken from the carotid artery of the mice by ether anesthesia (**Figure 1B**). The liver tissue was fixed in 10% formalin or kept at -80°C for subsequent analysis.

### Cell Culture and Treatment

AML-12 cell, an immortalized mouse hepatocyte, was generous gifts from Professor by Dr. Jung Joon Lee of Korea Research Institute of Bioscience and Biotechnology (Daejeon, Korea). AML-12 was incubated in DMEM supplemented with 10% fetal bovine serum (FBS), 100 U/ml penicillin and 100 mg/ml streptomycin at 37°C under 5% CO_2_. The cultures were passaged by trypsinization every 3 days and cells were plated in 100 mm culture dishes at a density of 1 × 10^6^ cells per dish in DMEM. For the experiment, cells were pretreated with AA (5, 10, 20 μM), or AICAR (500 μM), or GW3965 (1 μM), or SRT1720 (6 μM), or Nicotinamide (20 mM) for 2 h, respectively, and then following treated with EtOH (200 mM) and LPS (10 ng/ml) for additional 48 h.

### Serum Aminotransferase and Triglyceride Measurement

The blood samples were separated by centrifugation at 3000 rpm for 30 min at 4°C and levels of ALT and AST of the blood serums were measured using Arkraysp-4430 fully automatic chemistry analyzer. The serum TG levels were measured by TGs Assay kit.

### Histopathological Assay

Liver tissue was fixed in 10% formalin solution and embedded in paraffin and cut into 5 μm section. Then the sections were deparaffinized with xylene and gradient ethanol dehydration. Sections were stained with hematoxylin-eosin solution, and dehydrated with gradient alcohol and xylene and sealed with neutral gum. The histopathological sections were examined under light microscopy and evaluated scores from 0 to 5 (0 = no lesions; 1 = minimal lesions; 2 = mild lesions; 3 = moderate lesions; 4 = marked lesions; and 5 = severe lesions) (Leung et al., 2013).

### Immunohistochemistry Analysis

Sections were incubated with 5% Goat Serum for 30 min in a humidified chamber at room temperature and blocking with Antibody-Ms-mAb-to-SREBP-1 (Abcam, Cambridge, MA, USA) at 37°C for 3 h. Then the sections were incubated with Biotin-SP-Conjugated Affinipure Fab Fragment Goat Anti-Mouse IgG for 20 min at room temperature. HRP labeled secondary antibody were incubated according to the steps mentioned in the statement of the MaxVision^TM^ HRP-Polymer anti-mouse/rabbit IHC kit (Maixin Biol, Fu Zhou, China). The tissue slides were counterstained 10–15 s with hematoxylin, dehydrated gradient alcohol and xylene, and sealed with neutral gum in the end.

The immunohistochemical staining was analyzed with Image Pro-Plus 6.0, expressed in mean optical density (MOD). Four fields of vision without any overlap in each section were selected randomly and photographed with 200 magnifications. It was required to detect the area of valid measure-area by software firstly, and then measured the integrated optic density of targeting stain region in this area. The percent of integrated optic density of the area was MOD (Wan et al., 2010).

### Oil Red O Staining Analysis

Cultivated cell dishes were washed in deionized water and fixed in ice-cold 60% isopropanol quickly, and then blocking with oil red O solution for 10 min at room temperature, then washed with deionized water and stained with hematoxylin. In the end, tissue sections were sealed with glycerin gelatin. The oil red O staining was analyzed with Image Pro-Plus 6.0, expressed in MOD (Wan et al., 2010).

### Immunofluorescence Staining

The frozen sections were fixed in the ice-cold mixed liquor of acetone and methanol, dried, dehydrated, and blocked by 5% goat serum. The sections were incubated with the primary antibody against F4/80 (Abcam, Cambridge, MA, USA) at room temperature for 2 h. Then, the sections were incubated with Alexa Fluor Goat pAb to Rat IgG (Abcam, Cambridge, MA, USA) at room temperature for 1 h. Incubated with DAPI, the sections were visualized under microscope. The immunofluorescence staining was analyzed with Image Pro-Plus 6.0, expressed in MOD (Wan et al., 2010).

### Western Blotting Analysis

Protein isolation and western blot analysis were performed as described in literature (Wu et al., 2010). Briefly, protein samples were placed in sodium dodecyl sulfate-polyacrylamide gel electrophoresis and blotted onto polyvinylidene difluoride membranes. The membranes were blocking with non-fat dry milk for 1 h and incubated at room temperature for 2 h with p-LKB1, LKB1, p-AMPKα, AMPKα, p-AMPKβ, AMPKβ, p-ACC, ACC (Cell Signaling Technology, Boston, MA, USA) or SREBP-1, CYP2E1, GAPDH, Sirt1, PPARγ (Abcam, Cambridge, MA, USA) or caspase-1, β-actin, PPARα (Santa Cruz, CA, USA) or IL-1β (R&D Systems Europe Ltd., Abingdon, UK). Then the membranes were followed by incubated with HRP-conjugated secondary antibody for 1 h at room temperature and visualized by ECL Prime Western Blotting Detection Reagent (Bio-Rad, USA).

### RT-PCR Assay

Total RNA was extracted from mouse liver tissues by Eastep total RNA Extraction Kit (Beyotime Institute of Biotechnology, China) according to the manufacturer’s protocol. cDNA was prepared using 500 ng of total RNA, carried out on the ABI Veriti^®^ Thermal Cyclers. The primer sequences used in PCR are shown in **Table 1**. PCR products were run on a 2% agarose gel and ethidium bromide. The mRNA expression level of endogenous GAPDH was used as an internal. The primer sequences used in PCR are shown in **Table 1**.

**Table 1:** The primer sequences for RT-PCR.

Target genes	Primer sequences	Product length (bp)
IL-1β	5′-TCTTTGAAGAAGAGCCCATCC-3′	61
	5′-CTAATGGGAACGTCACACAC-3′	
SREBP-1	5′-CTTAGCCTCTACACCAACTG-3′	246
	5′-AGGAATACCCTCCTCATAGC-3′	
IL-18	5′-GATCAAAGTGCCAGTGAACC-3′	233
	5′-AACTCCATCTTGTTGTGTCC-3′	
LXRβ	5′-CACCATTGAGATCATGTTGC-3′	309
	5′-TTGATCCTCGTGTAGGAGAG-3′	
LXRα	5′-CCTCTGGCTTCCATTACAAC-3′	201
	5′-CTTCTGACAGCACACACTC-3′	
GAPDH	5′-ATGGTGAAGGTCGGTGTGAA-3′	233
	5′-CGCTCCTGGAAGATGGTGAT-3′	


### Statistical Analyses

All data were expressed as the mean ± SD statistical differences. Statistical analyses were performed using one-way analysis of variance (ANOVA) and Tukey’s multiple comparison tests. Statistically significant differences between groups were defined as *p* < 0.05. Calculations were performed using GraphPad Prism (GraphPad Software, San Diego, CA, USA).

## Results

### AA Effectively Attenuated Alcohol Exposure-Induced Fatty Liver

**FIGURE 2:**
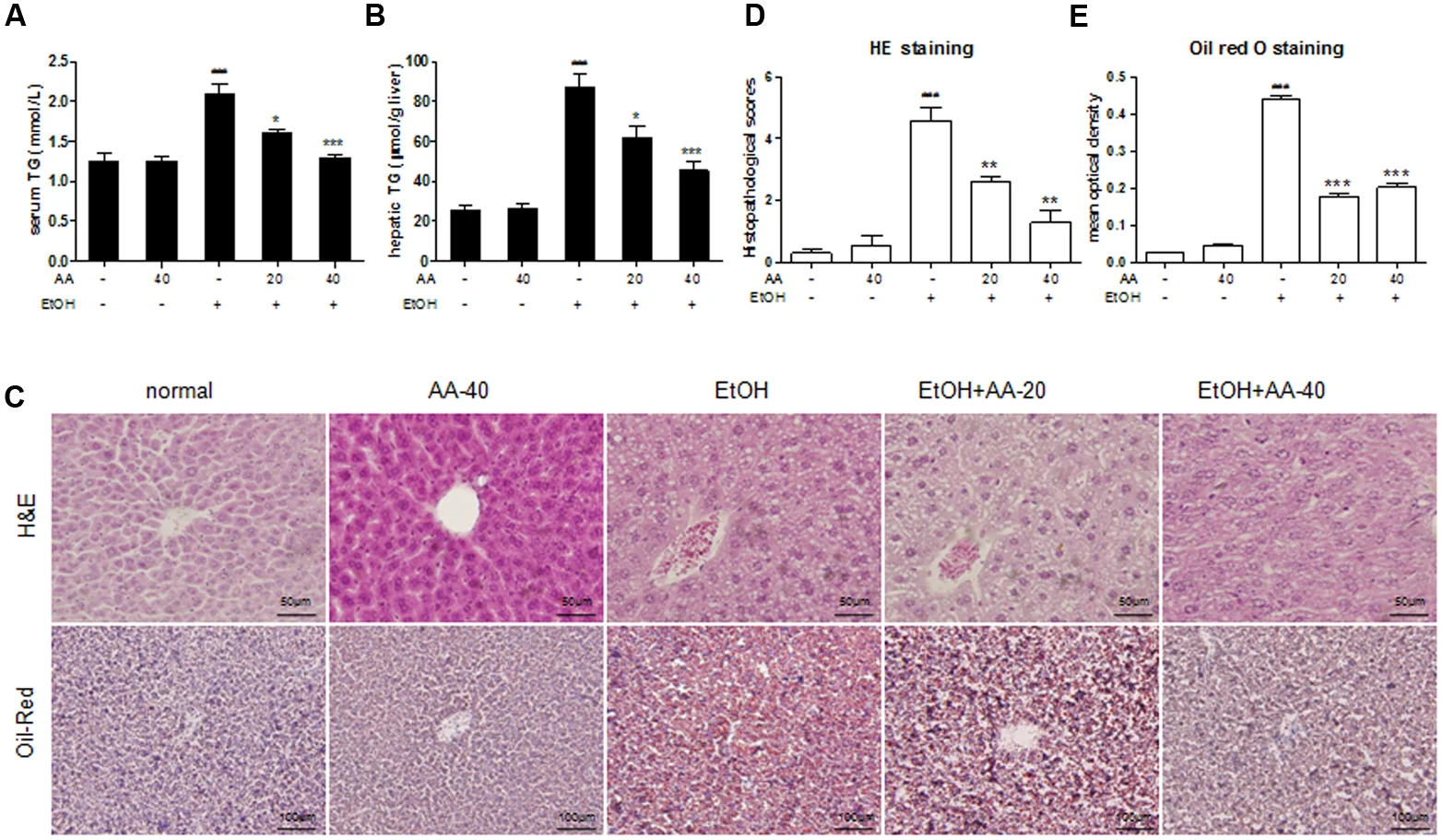
**Effects of AA on the serum and hepatic TG levels and hepatic morphology.** **(A)** Serum TG levels; **(B)** hepatic TG levels; **(C)** Hematoxylin and Eosin (H&E) stain, and oil red O stain. HE was calculated histopathological scores **(D)** and Oil red O staining **(E)** was analyzed with Image Pro-Plus 6.0. Data were presented as mean ± SD (*n* = 6). ^###^*p* < 0.001 vs. normal group; ^∗^*p* < 0.05, ^∗∗∗^*p* < 0.001 vs. EtOH group.

Mice were given ethanol (5 g/kg) in 24 h to simulate a binge drinking. Acute ethanol challenge induced significant increase of the serum ALT and AST activities, and also led to significant increase of serum LDH and ALP, which present the same clinical meaning with ALT/AST. And all these serum parameters were suppressed by AA pretreatment (**Figures 1C–E**). And AA pretreatment also reversed increased serum TG and hepatic TG levels after acute ethanol challenge (**Figures 2A,B**). In consistent with above parameters, acute ethanol challenge led to massive steatosis in mice liver tissue, which was obviously alleviative in AA pretreatment group suffering ethanol (**Figures 2C,D**). In the liver section of ethanol group, red-stained lipid droplets were observed in oil red staining. However, no obvious fat droplets were observed in the liver section of AA pretreatment group suffering ethanol (**Figures 2C,E**). These results indicate that AA has hepatic protective effects against alcoholic liver injury after binge drinking.

### AA Pretreatment Suppressed SREBP-1 and CYP2E1 Activations Induced by Ethanol

**FIGURE 3:**
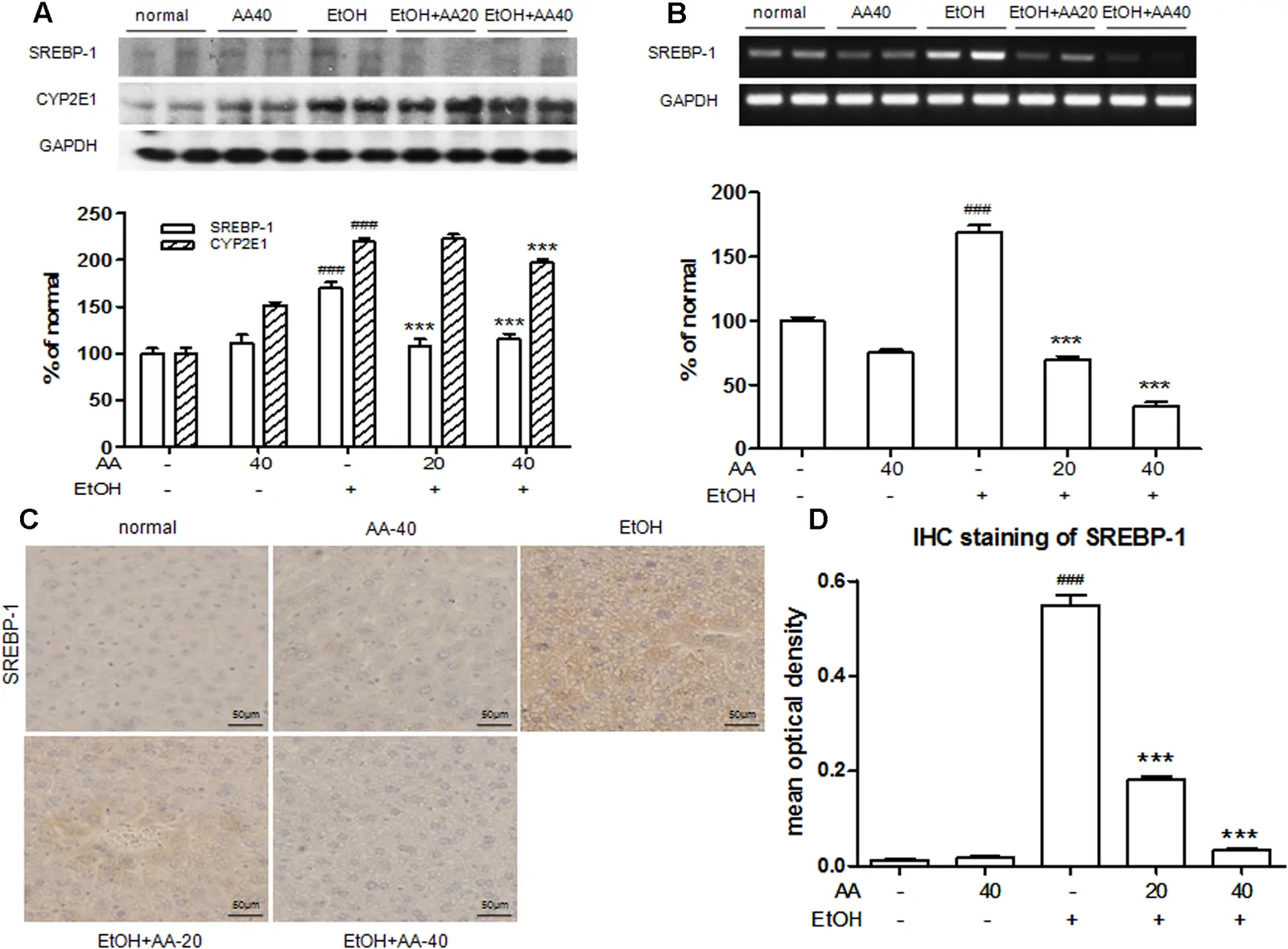
**Acanthoic acid pretreatment suppressed SREBP-1and CYP2E1 activations induced by ethanol.** **(A)** Western blot analysis of SREBP-1 and CYP2E1 protein expressions after ethanol challenge; **(B)** RT-PCR analysis of SREBP-1 mRNA expression after ethanol challenge; **(C)** Immunohistochemical staining of SREBP-1 after ethanol challenge. **(D)** Immunohistochemical staining of SREBP-1 was analyzed with Image Pro-Plus 6.0. ^###^*p* < 0.001 vs. normal group; ^∗∗∗^*p* < 0.001 vs. EtOH group.

Sterol regulatory element binding protein 1 is an important nuclear transcription factor involved in fatty acid synthesis, which mediates lipogenesis pathway. To investigate the effect of AA on SREBP-1, the protein and mRNA levels of SREBP-1 were detected. As shown in **Figures 3A,B**, acute ethanol challenge increased SERBP-1 protein and mRNA levels compared to the normal mice. These changes were significantly reversed by AA pretreatment. And no significant change of SREBP-1 was observed in only AA pretreatment group. Immunohistochemical detection of SREBP-1 levels also showed an increase by binge ethanol treatment with brown positive stained (**Figures 3C,D**). AA pretreatment decreased SREBP-1 positive expressions caused by ethanol. And almost no positive stained in normal and only AA groups. CYP2E1 is one central pathway in the ability of ethanol to induced lipid peroxidation and oxidative stress. In **Figure 3A**, CYP2E1 protein levels were increased after ethanol challenge, and were significantly suppressed with AA (40 mg/kg) pretreatment. AA pretreatment could attenuate binge ethanol-induced oxidative stress via CYP2E1 suppression.

### AA Pretreatment Ameliorated Hepatic Inflammation in Acute Alcohol Exposure Mice

**FIGURE 4:**
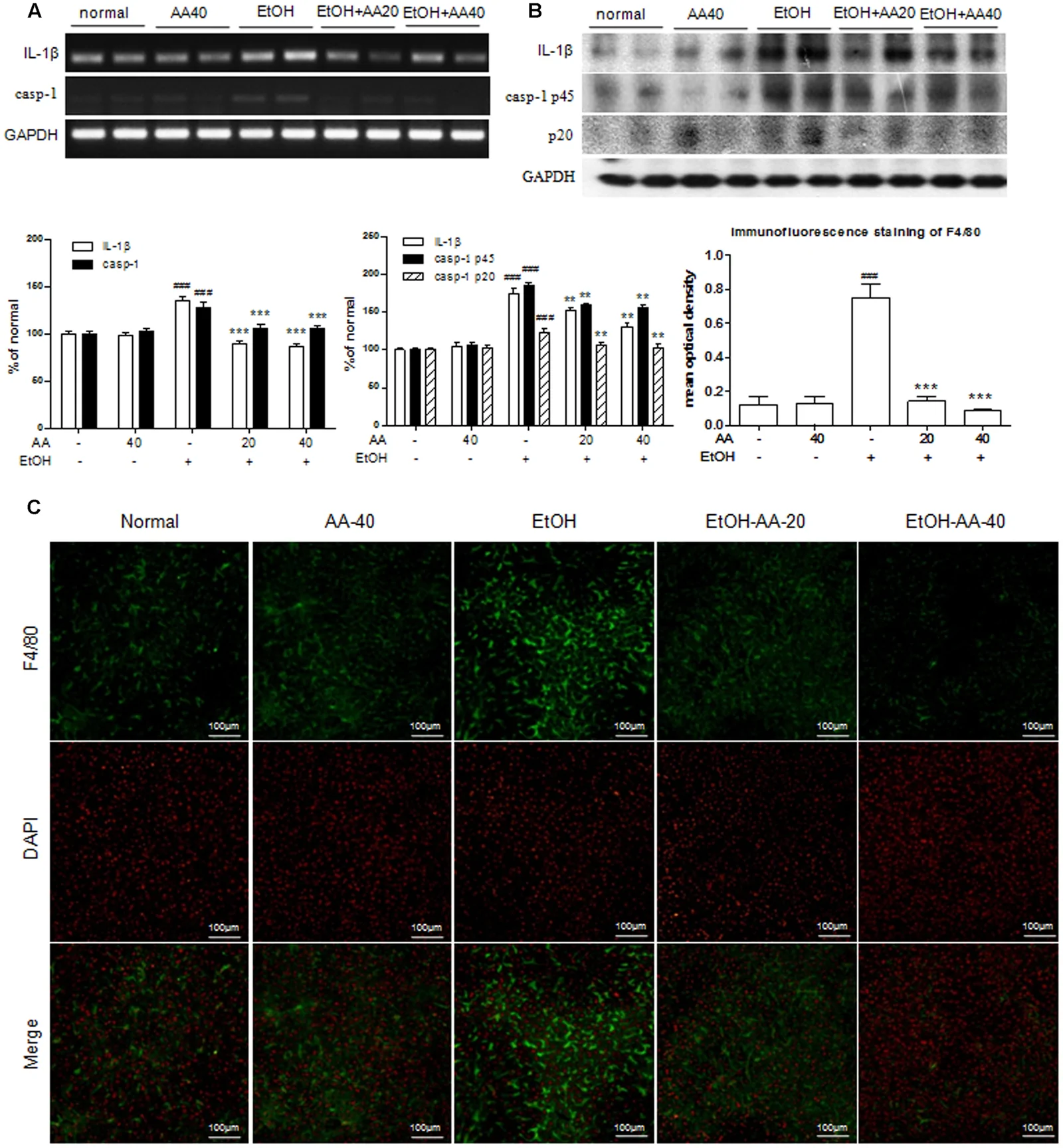
**Acanthoic acid pretreatment ameliorated hepatic inflammation in acute alcohol exposure mice.** **(A)** RT-PCR analysis of IL-1β and caspase-1 mRNA expressions after ethanol challenge; **(B)** Western blot analysis of IL-1β and caspase-1 protein expressions after ethanol challenge; **(C)** Immunofluorescence staining analysis of F4/80 expression in the livers of mice. Immunofluorescence staining of F4/80 was analyzed with Image Pro-Plus 6.0. ^###^*p* < 0.001 vs. normal group; ^∗∗^*p* < 0.01, ^∗∗∗^*p* < 0.001 vs. EtOH group.

Liver immune cells, such as Kupffer cells, play a significant role in ethanol challenge. Ethanol exposure stimulates gut to release LPS, and then produce pro-inflammatory cytokines in response to LPS. IL-1β, one of the major caspase-1 targets, is a pro-inflammatory cytokine that is cleaved by caspase-1 to yield a 17 kDa mature form (Ghiringhelli et al., 2009). With ethanol challenge, the IL-1β protein and mRNA levels were significantly increased compared to that in the normal group. The up-regulations of IL-1β were markedly inhibited with AA pretreatment (**Figures 4A,B**). Caspase-1 regulates the inflammatory response by promoting the processing and release of inflammatory factors. AA pretreatment reversed caspase-1 protein and mRNA levels that increased by ethanol challenge (**Figures 4A,B**), which also demonstrated that AA inhibited caspase-1 to block the processing and release of pro-inflammation cytokines. Expressions of F4/80, a cell surface glycoprotein and expressed in mature macrophages, in mice liver were detected by immunofluorescence staining. The expressions of F4/80 (green) were markedly observed in ethanol group, and AA pretreatment decreased the F4/80 expressions (**Figure 4C**). These results demonstrated that AA could suppress pro-inflammatory cytokines produced by mature macrophage underlying ethanol exposure.

### Effects of AA and Acute Ethanol Challenge on the Sirt1 Pathway and the Phosphorylation of LKB1, AMPK and ACC

**FIGURE 5:**
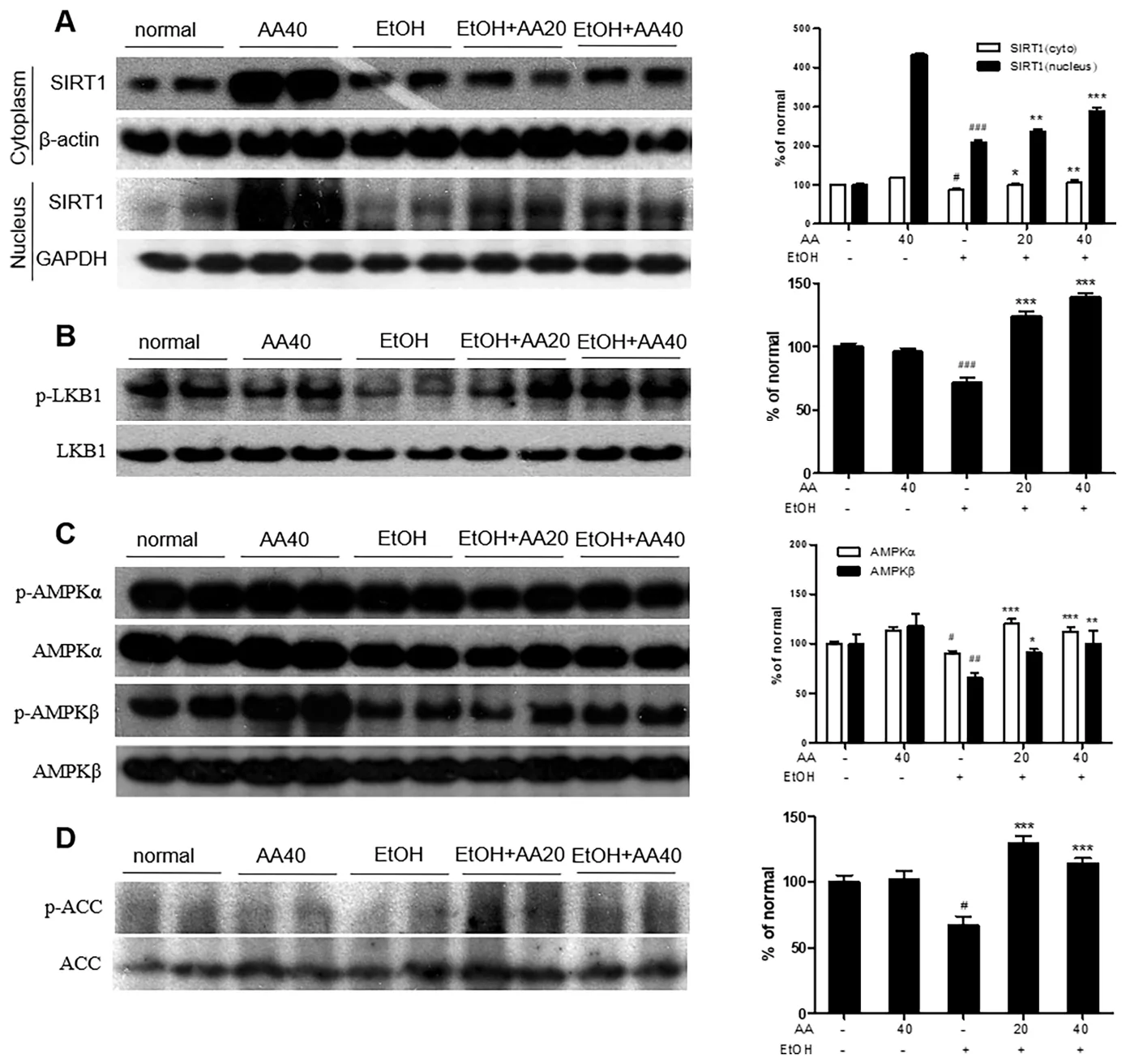
**Effects of AA and acute ethanol challenge on the Sirt1 pathway and the phosphorylation of LKB1, AMPK and ACC.** **(A)** Representative western blot analysis of Sirt1 in the cytoplasm and the nucleus was normalized based on the internal control β-actin or GAPDH; **(B)** Representative western blot analysis of p-LKB1 and total LKB1; **(C)** Representative western blot analysis of p-AMPKα, p-AMPKβ, and total AMPKα and AMPKβ; **(D)** Representative western blot analysis of p-ACC and total ACC. ^#^*p* < 0.05, ^##^*p* < 0.01,^###^*p* < 0.001 vs. normal group; ^∗^*p* < 0.05, ^∗∗^*p* < 0.01, ^∗∗∗^*p* < 0.001 vs. EtOH group.

Sirtuin 1 is a longevity gene and regulates lipid homeostasis, while lipid homeostasis the critical reasons in the development of fatty liver caused by alcohol. As shown in **Figure 5A**, expressions of Sirt1 in the cytoplasm and the nucleus were decreased by ethanol challenge. AA pretreatment increased Sirt1 expressions in the cytoplasm and the nucleus. Especially, single AA treatment obviously increased Sirt1 in the cytoplasm and the nucleus, which indicated that AA might be a potential Sirt1 agonist. AMPK is a major regulator of lipid and glucose metabolism. Thus we investigated whether the protective effect of AA was related with AMPK activation and it’s upstream or downstream, such as LKB1 or ACC, against ethanol challenge. The results of western blot revealed p-LKB1, p-AMPKα, p-AMPKβ, and p-ACC levels were significantly decreased in the liver of ethanol group compared with those of normal group, while AA pretreatment showed obviously increased p-LKB1, p-AMPKα, p-AMPKβ, and p-ACC levels when compared with ethanol group (**Figures 5B–D**).

### Effects of AA and Acute Ethanol Challenge on the LXRs Pathway, PPARα and PPARγ Expressions

**FIGURE 6:**
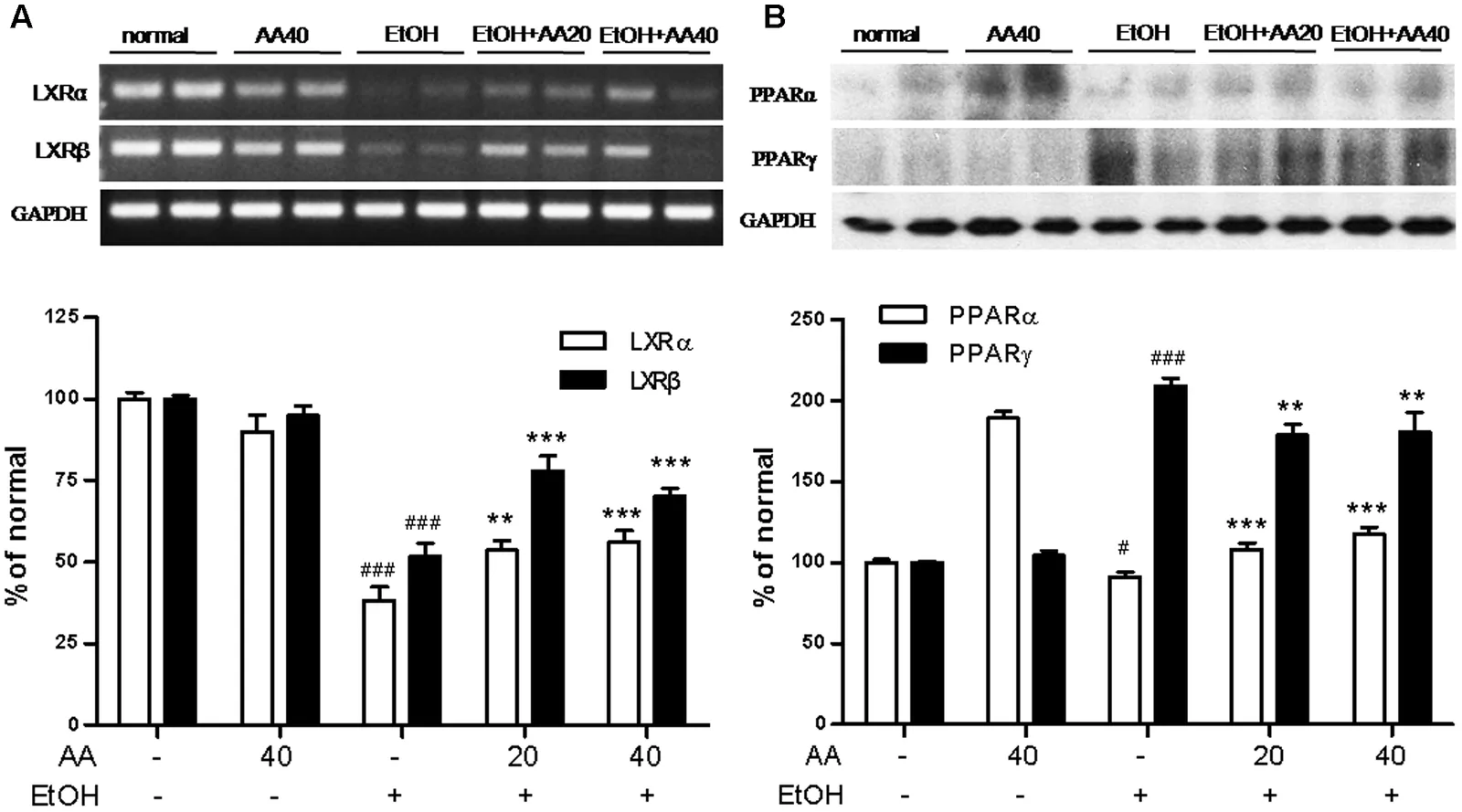
**Effects of AA and acute ethanol challenge on the LXRs pathway, PPARα and PPARγ expressions.** **(A)** RT-PCR analysis of LXRα and LXRβ was normalized based on the internal control GAPDH; **(B)** Representative western blot analysis of PPARα and PPARγ was normalized based on the internal control GAPDH. ^#^*p* < 0.05, ^###^*p* < 0.001 vs. normal group; ^∗∗^*p* < 0.01, ^∗∗∗^*p* < 0.001 vs. EtOH group.

Liver X receptors regulate cholesterol metabolism and hepatic fat metabolism. With ethanol challenge, LXRα and LXRβ mRNA levels were significantly decreased compared to normal group, and AA markedly reversed LXRα and LXRβ mRNA levels (**Figure 6A**). PPARα and PPARγ are the members of the ligand-activated nuclear receptor transcription factor superfamily, and participate in lipogenesis, energy and glucose homeostasis. As shown in **Figure 6B**, the protein levels of PPARα were decreased by ethanol challenge compared with those of the normal group. AA pretreatment restored the protein levels of PPARα compared with those of ethanol group (**Figure 6B**). Interestingly, ethanol challenge increased the protein levels of PPARγ compared with normal group, while AA pretreatment decreased the protein levels of PPARγ (**Figure 6B**). However, this result is similar with overexpression of PPARγ in hepatocytes further leads to the development of lipogenic steatosis (Rull et al., 2014).

### AA Increases the LXRs Activities and Activates the Sirt1/LKB1/AMPK/ACC Signaling Pathways in EtOH/LPS Stimulated AML-12 Cell

**FIGURE 7:**
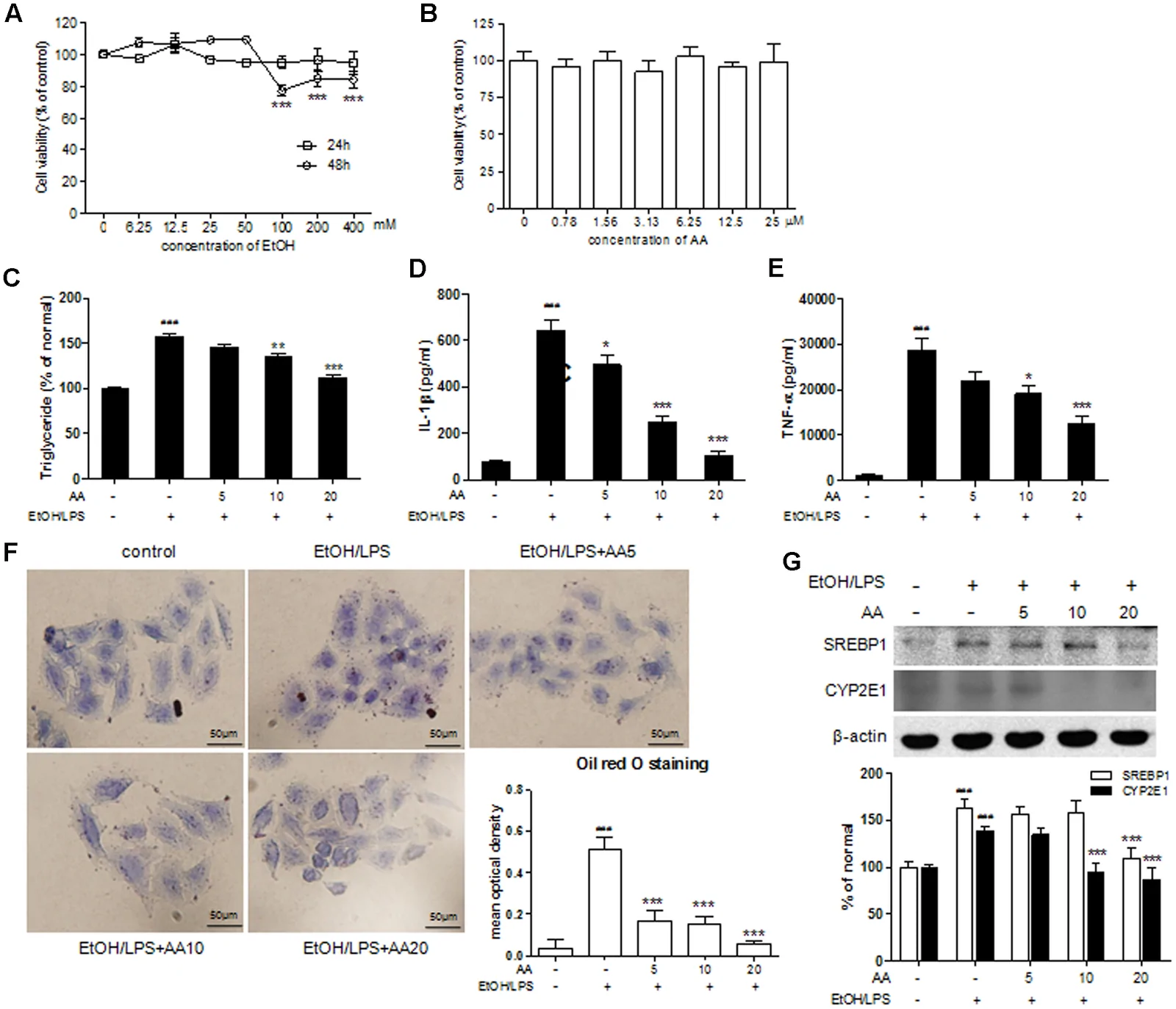
**Acanthoic acid ameliorated lipid deposition and inflammation in EtOH/LPS stimulated AML-12 cells.** **(A)** Cell viability of EtOH on AML-12. **(B)** Cell viability of AA on AML-12; **(C)** Cell TG levels; **(D)** Cell IL-1β levels; **(E)** Cell TNF-α levels; **(F)** cell oil red O staining, and Oil red O staining was analyzed with Image Pro-Plus 6.0; **(G)** Representative Western blot analysis of SREBP1 and CYP2E1 was normalized based on the internal control β-actin. ^###^*p* < 0.001 vs. normal group; ^∗∗^*p* < 0.01, ^∗∗∗^*p* < 0.001 vs. EtOH group.

To clarify the relationship of SIRT1-LXR and LKB1/AMPK/ACC signaling on the regulation of alcohol exposure-induced liver lipid deposition and inflammation, we detected the relative signaling proteins under AA treatment in EtOH/LPS activity of HSCs. As in the animal experiments, ethanol challenge induced release of pro-inflammatory cytokines; ethanol and LPS were treatment in AML-12 cells to simulate ethanol-exposure mice. Firstly, we detected the effects of ethanol (0–400 mM) on the cell viability of AML-12 for 24 or 48 h (**Figure 7A**). And ethanol (100, 200, and 400 mM) for 48 h significantly decreased cell viability compared with that for 24 h. In **Figure 7B**, the cell viability of AA on AML-12 for 6 h were detected, and AA (0–25 μM) showed no significantly effects on cell viability of AML-12. Thus, we selected AA (5, 10, and 20 μM) for 2 h and following with ethanol (200 mM)/LPS (10 ng/ml) for 48 h to simulate animal experiments and researched the relative signaling pathway. In **Figures 7C–E**, TG, IL-1β, and tumor necrosis factor-α (TNF-α) levels in AML-12 cell were increased by treatment of EtOH/LPS, and reversed with AA treatment (10 and 20 μM for TG and TNF-α levels; 5, 10, and 20 μM for IL-1β levels). Red-stained lipid droplets in EtOH/LPS group were observed in oil red O staining. However, no obvious fat droplets were presented in AA treatment groups (**Figure 7F**). Expressions of SREBP-1 and CYP2E1were significantly increased with EtOH/LPS treatment, and AA decreased the expressions of SREBP-1 and CYP2E1 (**Figure 7G**).

**FIGURE 8:**
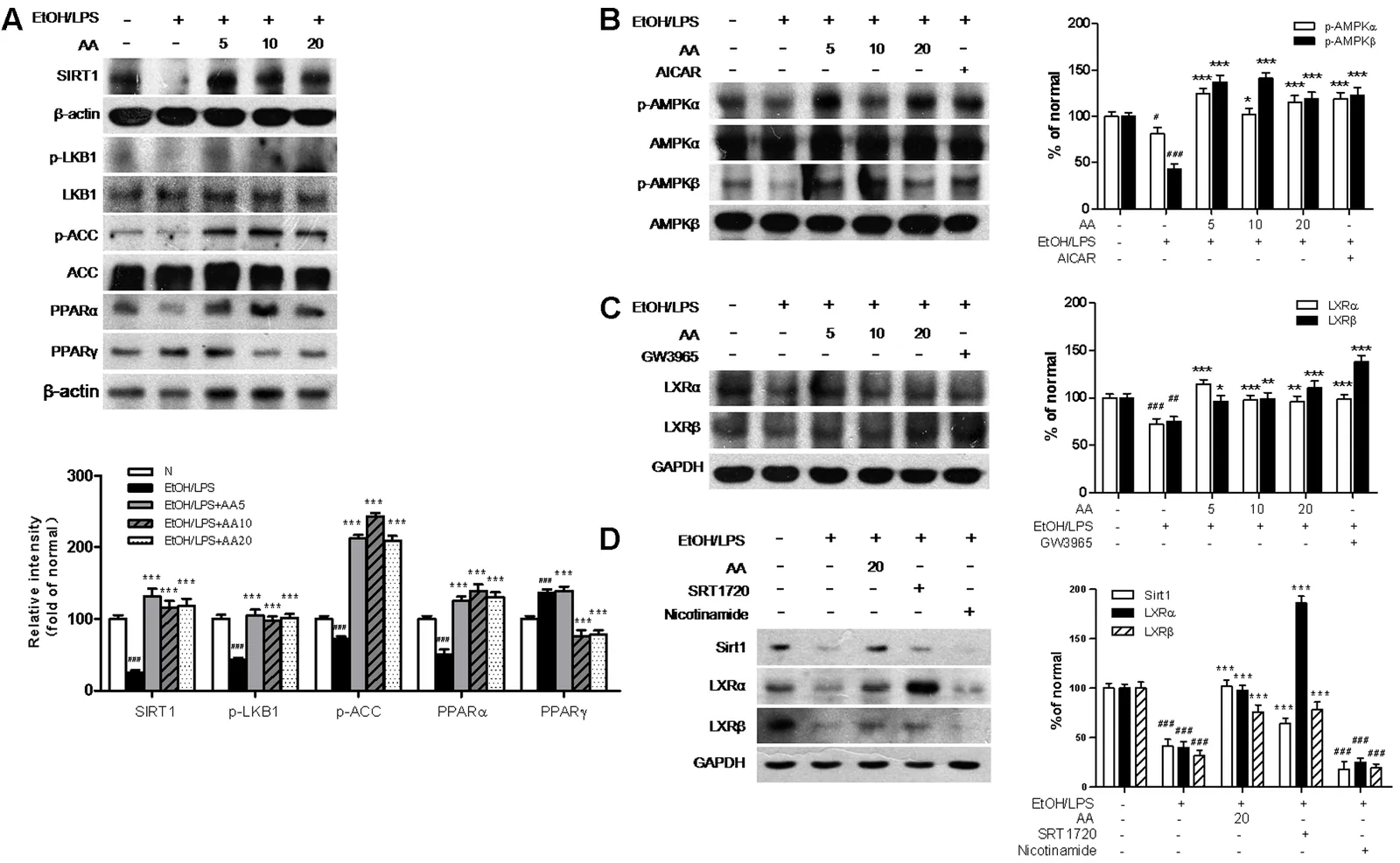
**Acanthoic acid increases the LXRs activities and activates the Sirt1/LKB1/AMPK/ACC signaling pathways in EtOH/LPS stimulated AML-12 cell.** AML-12 cells were pretreated with AA (5, 10, 20 μM), or AICAR (500 μM), or GW3965 (1 μM), or SRT1720 (6 μM), or Nicotinamide (20 mM) for 2 h, respectively, and then following treated with EtOH (200 mM) and LPS (10 ng/ml) for additional 48 h. **(A)** Representative western blot analysis of Sirt1, p-LKB1, p-ACC, PPARα, and PPARγ was normalized based on the internal control β-actin. **(B)** Representative western blot analysis of p-AMPKα, p-AMPKβ, total AMPKα and AMPKβ. **(C)** Representative western blot analysis of LXRα and LXRβ was normalized based on the internal control GAPDH. **(D)** Representative western blot analysis of Sirt1, LXRα and LXRβ was normalized based on the internal control GAPDH. ^#^*p* < 0.05, ^##^*p* < 0.01, ^###^*p* < 0.001 vs. normal group; ^∗^*p* < 0.05, ^∗∗^*p* < 0.01, ^∗∗∗^*p* < 0.001 vs. EtOH group.

In the signaling pathway, EtOH/LPS significantly decreased Sirt1, p-LKB1, p-ACC, PPARα protein expressions, and increased PPARγ protein expression, which were reversed with AA treatment (**Figure 8A**). And the regulation of AA on SIRT1-LXR and LKB1/AMPK/ACC signaling in AML-12 cells were similar with those in mice model. In order to verify the effects of AA on AMPK, LXRs, and Sirt1, we compared the effects of AA and above signaling agonists or inhibitor. In **Figure 8B**, AA increased p-AMPKα and p-AMPKβ protein expression as similar to AICAR, an agonist of AMPK. And AA also increased LXRα and LXRβ protein expressions as similar to GW3965, an agonist of LXRs (**Figure 8C**). AA increased Sirt1 protein expressions as similar to SRT1720, an agonist of LXRs; while shown a negative regulation compared with Nicotinamide, an inhibitor of LXRs (**Figure 8D**).

The results in AML-12 cell demonstrated AA increased Sirt1-LXRs and regulates LKB1/AMPK/ACC signaling to ameliorate alcohol exposure-induced liver lipid deposition and inflammation.

## Discussion

In current study, AA obviously ameliorated alcohol exposure-induced liver lipid deposition and inflammation by decreasing TG levels, SREBP-1 expressions, fats in liver tissue, IL-1 and IL-18 levels and caspase-1 expressions. Moreover, in alcohol exposure, LKB1/Sirt1/AMPK/ACC and LXRs pathways were necessary for the inhibition of AA on lipid deposition and inflammation. Therefore, the results supported a novel effect of AA on regulation of fatty acid homeostasis *via* Sirt1 signaling pathway, which suggested that AA would be a promising therapeutic agent for ameliorating the development of ALD.

Alcoholic liver diseases have become a major public health issue all over the world. Most people know that excessive drinking is harmful for health. It’s a pity there still is an increasing number of patients with ALD. Therefore, it’s very important to seek a natural therapeutic drug with high efficiency and low toxicity, and explore underlying mechanisms and potential clinical utilities. In our previous study, AA showed a strong hepatoprotective effect against hepatic injury and hepatic fibrosis induced by D-GalN/LPS, acetaminophen, or carbon tetrachloride (CCl_4_) (Nan et al., 2008; Wu et al., 2010; Bai et al., 2014). We also found AA ameliorated hepatic fibrosis via regulation LXRs pathway. AA is considered as a potent activator of liver X receptors (LXRα and LXRβ) targeting the cholesterol transporters (Jung et al., 2007). While LXRs play important roles in energy homeostasis, and directly control hepatic cholesterol metabolic regulations (Ghaddab-Zroud et al., 2014). In the current study, we analyzed how LXR may crosstalk with other signaling pathway during the regulation of AA on lipid deposition and inflammation induced by alcohol exposure.

In the present study, AA increased the LXRs expressions compared with ethanol exposure group. And activation of LXRs by AA played an important role in lipid metabolism and anti-inflammatory signaling, which was verified by tissue staining and inflammatory markers. *In vitro*, AA showed the same function with GW3965, a synthetic LXR agonist, and activated LXRs expression. Thus, LXRs are considered as attractive targets in treatment of AA on metabolic disease aiming for integration of lipid metabolic and inflammatory. However, Sirt1 positively regulates LXRs deacetylation. LXR acetylation is significant when a single conserved lysine adjacent to activation domain, such as K432 or K433 in LXRs. Sirt1 promotes deacetylation and subsequent ubiquitination and interacts with LXR. Li et al. (2007) found loss of Sirt1 reduced LXR targeting proteins involved in lipid metabolism *in vivo*. Above information suggests that Sirt1 is involved in regulation of AA on alcohol exposure-induced liver lipid deposition and inflammation *via* LXRs signaling.

Sirtuin 1 regulates longevity effect leaded by the energy restriction, and is known as a longevity gene (Shahedur and Rezuanul, 2011). Sirt1 catalyzes Apurinic/apyrimidinic endonuclease-1, enhances DNA base excision repair activity, and generates a unique metabolite (Kim et al., 2016). Hepatic metabolic derangements are the critical reasons in the development of fatty liver caused by alcohol. Sirt1 regulates lipid homeostasis by working with its substrate, such as peroxisome proliferators-activated receptor α (PPARα), which mediates fatty acid β-oxidation (Sharples et al., 2015). The expression of PPARα positively correlates with the expression of Sirt1 (Avila et al., 2016). In alcohol-exposure mice, we also found AA enhanced hepatic Sirt1expression with increasing PPARα expression. In the meantime, AA decreased SREBP-1 in protein and mRNA levels, CYP2E1 expression, and serum and hepatic TG levels. SREBP-1 is the major transcription factor regulating relative genes of hepatic fatty acid and TG synthesis, and can up-regulate cytochrome p4502E1 (CYP2E1) expression via free fatty acids, further control hepatic lipid oxidative stress and lipid peroxidation. Over expression of CYP2E1 increases lipid deposition and inflammation, and fat metabolism disorder. Therefore, activation of Sirt1 by AA may be important for the prevention of metabolic derangements caused by alcohol exposure.

Accumulating studies have shown that AMP-activated protein kinase (AMPK) is considered as a central regulator on lipid metabolism and glucose homeostasis. Activated AMPK inhibits fatty acid synthesis and stimulates energy-producing pathways (Dagon et al., 2016). Synergistically activations of AMPK and Sirt1 play key roles in insulin sensitivity and glycemic control (Banerjee et al., 2016). LKB1, the upstream AMPK-kinase, undertakes cellular kinase activity of AMPK via phosphorylation and then the following downstream substrates (Hardie et al., 2012). However, LKB1 is deacetylated on lysine 48 by Sirt1, and migrates from the nucleus to cytoplasm, then leading to activation (Yang et al., 2014). Generally, Sirt1-dependent activation of LKB1 simulates AMPK activation by phosphorylation and establishes the Sirt1-LKB1-AMPK signaling axis (Lan et al., 2008). AMPK also function the upstream of Sirt1, and stimulate Sirt1 activity via NAD+ level (Cantó et al., 2009). The crosstalk of Sirt1 and AMPK present in a bidirectional positive-feedback, and follow with amplified stimulation for downstream signaling. We observed the activation of Sirt1 and LXRs by AA involves the upregulation of LKB1/AMPK/ACC signaling pathways. With activation of Sirt1 and LXRs, AA supplementation reversed p-LKB1, p-AMPK, and p-ACC levels decreased by alcohol administration, which inhibits SREBP-1 and regulates fatty acid synthesis. A number of studies have demonstrated that acute or chronic alcohol increases SREBP-1, accumulates TG, and reduces fatty acid oxidation. The present study strongly implicated AA as a potent agonist of Sirt1-LXRs activation in alcohol exposure-induced liver lipid deposition and inflammation.

Alcoholic liver diseases generally progress with inflammation of liver (Cannon et al., 2016). Our study found F4/80, a mature macrophage marker, was observed with ethanol challenge. IL-1β and caspase-1 protein and mRNA expressions were also increased with ethanol challenge. While AA treatment significantly inhibited the expressions of F4/80, protein and mRNA expressions of IL-1β and caspase-1.These results indicated that ethanol challenge resulted in inflammation in the liver of mice. AA pretreatment significantly reversed these inflammation expressions, which demonstrated that AA could regulate inflammation caused by ethanol challenge.

**FIGURE 9:**
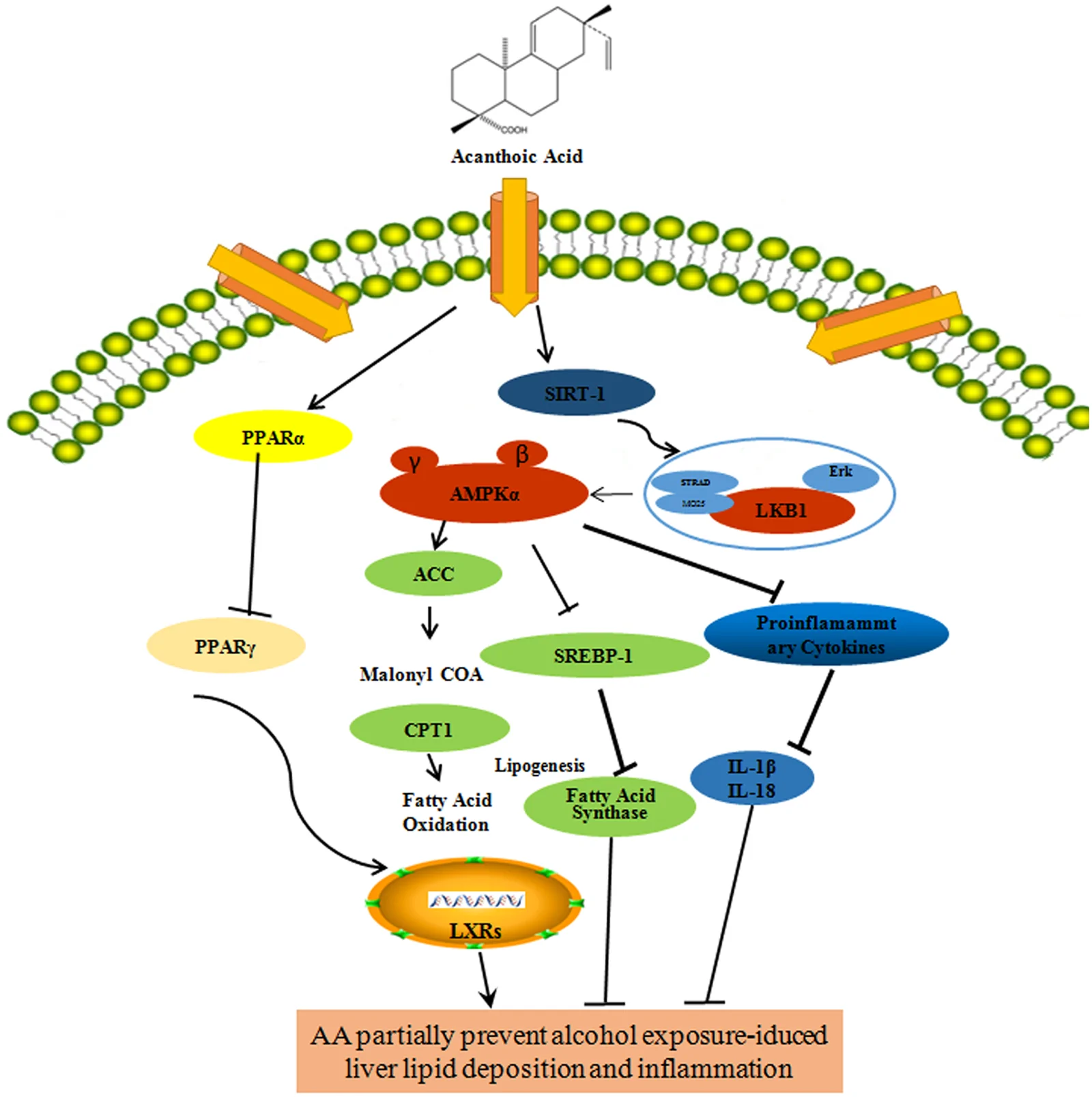
**A schematic representation showing the partially prevention of AA against liver lipid deposition and inflammation induced by alcohol**.

Given the effect of AA on the expression of Sirt1-LXRs and regulation of LKB1/AMPK/ACC, we concluded that AA increased Sirt1-LXRs, PPARα, reduced SREBP-1, CYP2E1 and PPARγ, cleaved caspase-1 induction, and consequently reduced IL-1β and increased IL-18 secretion through LKB1/AMPK/ACC pathways in alcohol-induced lipid deposition and inflammation (**Figure 9**). The study suggested that AA would provide a preventive therapeutic candidate for lipid deposition and inflammation in ALD and show advantages for potential clinical applications.

## Author Contributions

Y-LW and J-XN contributed to the design of the study, acquisition of data, and analysis and interpretation of the data. All authors contributed to the acquisition of data and analysis and interpretation of the data. All authors also participated in drafting or revising the manuscript and approved the final version of the manuscript for submission.

## Conflict of Interest Statement

The authors declare that the research was conducted in the absence of any commercial or financial relationships that could be construed as a potential conflict of interest.

## Abbreviations

ACC: Acetyl CoA carboxylase
ALP: alkaline phosphatase
ALT: alanine aminotransferase
AMPK: adenosine monophosphate-activated protein kinase
AST: aspartate aminotransferase
CYP2E1: Cytochrome P4502E1
IL-1β: Interleukin-1β
LDH: lactate dehydrogenase
LKB-1: liver kinase B-1
LPS: lipopolysaccharide
LXRs: liver X receptors
PPARα: peroxisome proliferator activated receptor α
PPARγ: peroxisome proliferator activated receptor γ
SIRT1: sirtuin 1
SREBP-1: sterol regulatory element binding protein 1
TG: triglyceride
