# Correction: Acanthoic acid can partially prevent alcohol exposure-induced liver lipid deposition and inflammation

**DOI:** 10.3389/fphar.2026.1947288

**Published:** 2026-09-23

**Authors:** You-Li Yao, Xin Han, Zhi-Man Li, Li-Hua Lian, Ji-Xing Nan, Yan-Ling Wu

**Affiliations:** 1 Key Laboratory for Natural Resource of Changbai Mountain and Functional Molecules, Ministry of Education, College of Pharmacy, Yanbian University, Yanji, China; 2 Clinical Research Center, Yanbian University Hospital, Yanji, China

**Keywords:** acanthoic acid, alcohol, liver lipid deposition, inflammation, Sirt1, LXRs

In the published article, there was an error in Figures 5A,C as published. The incorrect images were erroneously inserted.

**FIGURE 5:**
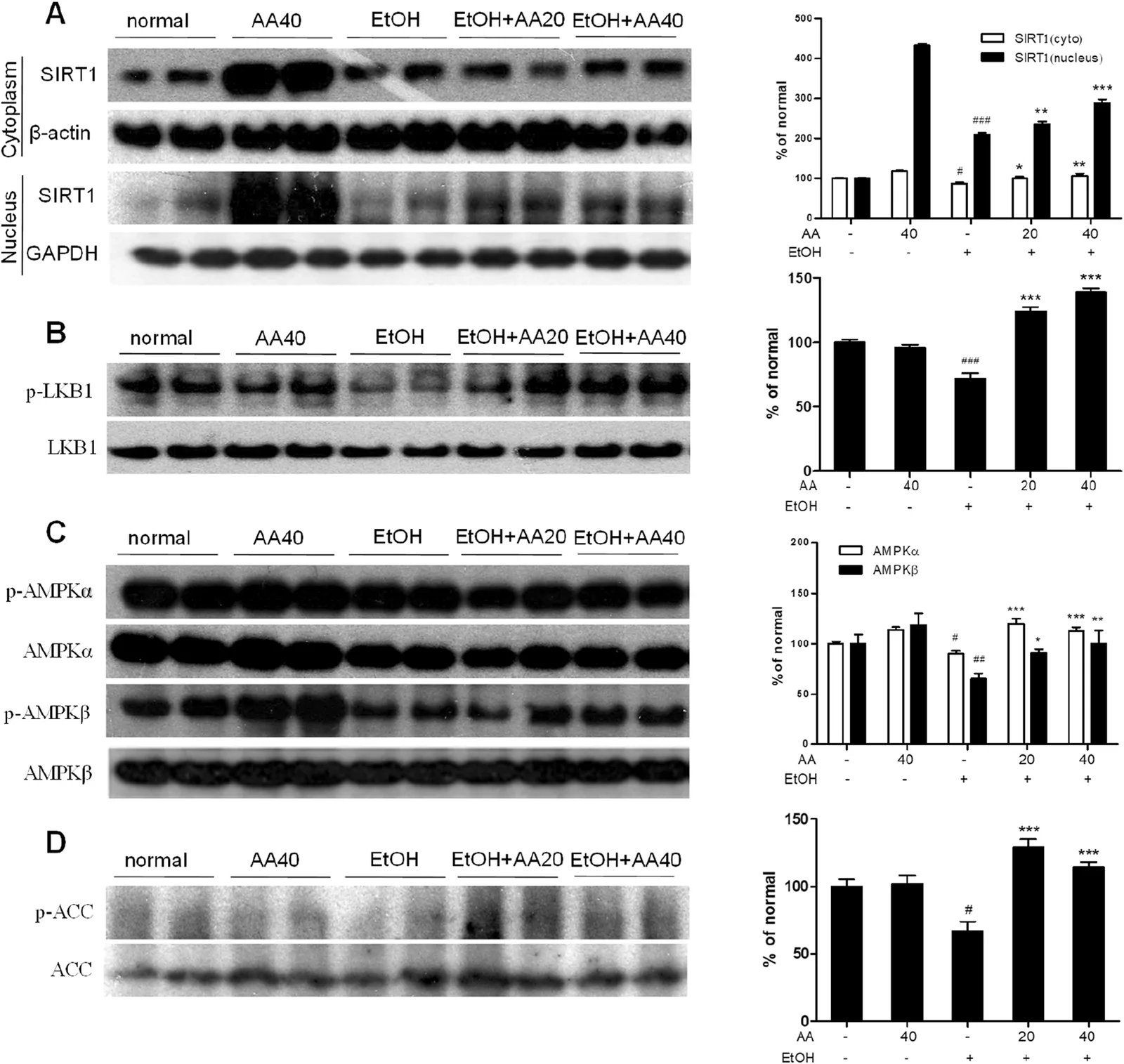
Effects of AA and acute ethanol challenge on the Sirt1 pathway and the phosphorylation of LKB1, AMPK and ACC. **(A)** Representative Western blot analysis of Sirt1 in the cytoplasm and the nucleus was normalized based on the internal control β-actin or GAPDH; **(B)** Representative Western blot analysis of p-LKB1 and total LKB1; **(C)** Representative Western blot analysis of p-AMPKα, p-AMPKβ, and total AMPKα and AMPKβ; **(D)** Representative Western blot analysis of p-ACC and total ACC. ^#^p < 0.05, ^##^p < 0.01, ^###^p < 0.001 vs. normal group; *p < 0.05, **p < 0.01, ***p < 0.001 vs. EtOH group.

Specifically, in Figure 5A, the blot of β-actin image was mistakenly shown, due to the carelessness of the picture combination and image processing. In Figure 5C, the blot of AMPKα was inadvertently displayed as the p-AMPKα.

The corrected Figure 5 and their captions appear below.

The original article has been updated.

